# Insight into the Mechanical Performance of the UHPC Repaired Cementitious Composite System after Exposure to High Temperatures

**DOI:** 10.3390/ma14154095

**Published:** 2021-07-22

**Authors:** Qing Chen, Zhiyuan Zhu, Rui Ma, Zhengwu Jiang, Yao Zhang, Hehua Zhu

**Affiliations:** 1 Key Laboratory of Advanced Civil Engineering Materials, Tongji University, Ministry of Education, 4800 Cao’an Road, Shanghai 201804, China; chenqing19831014@163.com (Q.C.); jzhw@tongji.edu.cn (Z.J.); 2 School of Materials Science and Engineering, Tongji University, Shanghai 201804, China; 15900737182@163.com (Z.Z.); 12012093@zju.edu.cn (R.M.); 3 Structural Health Monitoring and Control Key Laboratory of Hebei Province, Shijiazhuang Tiedao University, Shijiazhuang 050043, China; 4 State Key Laboratory of Mechanical Behavior and System Safety of Traffic Engineering Structures, Shijiazhuang Tiedao University, Shijiazhuang 050043, China; 5 State Key Laboratory for Disaster Reduction in Civil Engineering, Tongji University, 1239 Siping Road, Shanghai 200092, China; zhuhehua@tongji.edu.cn

**Keywords:** ultra-high-performance concrete, cementitious composite system, high temperature, mechanical, microstructures

## Abstract

In this paper, the mechanical performance of an ultra-high-performance concrete (UHPC) repaired cementitious composite system, including the old matrix and the new reinforcement (UHPC), under various high temperature levels (20 °C, 100 °C, 300 °C, and 500 °C) was studied. In this system, UHPC reinforced with different contents of steel fibers and polypropylene (PP) fibers was utilized. Moreover, the physical, compressive, bonding, and flexural behaviors of the UHPC repaired system after being exposed to different high temperatures were investigated. Meanwhile, X-ray diffraction (XRD), baseline evaluation test (BET), and scanning electron microscope (SEM) tests were conducted to analyze the effect of high temperature on the microstructural changes in a UHPC repaired cementitious composite system. Results indicate that the appearance of the bonded system changed, and its mass decreased slightly. The average percentage of residual mass of the system was 99.5%, 96%, and 94–95% at 100 °C, 300 °C, and 500 °C, respectively. The residual compressive strength, bonding strength, and flexural performance improved first and then deteriorated with the increase of temperature. When the temperature reached 500 °C, the compressive strength, bonding strength, and flexural strength decreased by about 20%, 30%, and 15% for the UHPC bonded system, respectively. Under high temperature, the original components of UHPC decreased and the pore structure deteriorated. The cumulative pore volume at 500 °C could reach more than three times that at room temperature (about 20 °C). The bonding showed obvious deterioration, and the interfacial structure became looser after exposure to high temperature.

## 1. Introduction

Ultra-high-performance concrete (UHPC) generally exhibits high strength, reliability, ductility, energy dissipation, and good durability [1,2,3,4], as a result of which the application of UHPC can help reduce the consumption of cementitious materials and develop a sustainable society. Meanwhile, UHPC can show a high quality of bond with old concrete compared to conventional concrete. Therefore, using UHPC to reinforce or repair damaged specimens of in-service engineering structures has become appealing [5].

Several researchers have investigated the performances of a bonded repaired system composed of repair material UHPC and normal concrete (NC). Sarkar [6] studied the influence of surface roughness on the bonding compatibility between UHPC and the matrix, including the surface without any treatment, the surface with low roughness (0.04–0.09 inch), and the surface with high roughness (0.25 inch). He et al. [7] conducted experimental research on the mechanical performances of the bonded concrete systems with different roughness interfaces on the 180-day concrete matrix. Their results showed that appropriate surface roughening treatment was beneficial to the bonding compatibility of the system, and damage would occur at the interface without surface treatment. Hussein et al. [8] investigated the mechanical performance of the repaired system with 0%, 1%, 1.5%, and 2% fiber volume content in the UHPC layer. They observed that steel fiber could significantly improve the compressive strength and flexural capacity of the bonded system. Similar conclusions were also reached by Mahmud et al. [9] and Ren et al. [10]. In addition, many theoretical models were carried out on the weak link of the repaired system—the interfacial transition zone [11,12]. Xie et al. proposed a model of a bonded concrete system, which divided the bonding surface into a penetrating layer, a strongly-affected layer, and a weakly-affected layer [13]. Among them, the strongly-affected layer dominated the interface performance of the repaired system. Farzad et al. established a matrix–interface transition zone-repair material ternary system and introduced a calculation model for bonding strength [14].

Due to its dense structure, UHPC is more prone to explosive spalling when exposed to high temperature compared with the ordinary concrete [15,16]. Chen et al. [17] explored the mechanical properties of UHPC before and after exposure to high temperatures. They found that the mechanical properties increased slightly at the initial stage of fire. However, as the target temperature reached 500 °C, the residual flexural strength decreased significantly. Similar works could be found by Choe et al. [18]. Ozawa et al. [19] investigated the effects of various fibers on the high-temperature spalling in high-performance concrete. Their results revealed that the specimens with jute fiber, water-soluble polyvinyl alcohol (WSPVA) fiber, and PP fiber did not explosively spall. In addition, many researchers have reached a consensus on the role of organic fibers in reducing the explosive risk of UHPC [20,21,22,23].

From the above, the performance of UHPC material under fire or high temperature environment has been studied by many researchers. However, it is worth mentioning that there is little research on the behavior of UHPC bonded systems under high temperature, especially the interface behavior between the UHPC layer and the normal concrete layer. In order to better understand the thermal degradation of the composite system repaired by UHPC, this paper experimentally investigates the residual compressive strength and the flexural behavior of this composite system, as well as the bonding strength between the old matrix and the new reinforcement UHPC after exposure to different temperature conditions. Furthermore, microstructural variations have been observed by XRD, BET, and SEM to show some light on the action mechanism of high temperature. The rest of the paper is organized as follows: Section 2 illustrates the raw materials and the preparation of the bonded systems. Section 3 lists the experimental program and the testing methods. The experimental results and discussions are provided in Section 4. The last section gives the main conclusions for this study.

## 2. Materials and Sample Preparation

### 2.1. Materials

In this study, P.II 52.5 Portland cement provided by Jiangnan Onoda Cement Co., Ltd. was employed as the binder, and its chemical components are listed in Table 1. The silica fume was produced by Topken enterprise with 96.2% SiO_2_ content. The quartz powder was provided by Shengkuo Building Materials Co., Ltd., and its mean diameter was 12 μm. Quartz sand (particle size 0.15–0.6 mm) and river sand (fineness modulus 2.48) were used as fine aggregate. PC200 type superplasticizer provided by Inshine New Material Technology Co., Ltd. was used. Its water reducing rate was 23%. The properties of steel fibers and polypropylene fibers are presented in Table 2.

### 2.2. Sample Preparation

The UHPC repaired system studied in this paper consisted of two parts: the ordinary cement mortar matrix layer and the UHPC layer. The basic mix proportion of the matrix and UHPC is shown in Table 3. The mixing time for UHPC and matrix layer was about 15 min and 5 min, respectively. As displayed in Table 4, the bonded systems containing different fiber contents were studied, which are represented by Sf0, Sf1, Sf2, and Sf1pp1. Three specimens in each group were prepared.

**Figure 1:**
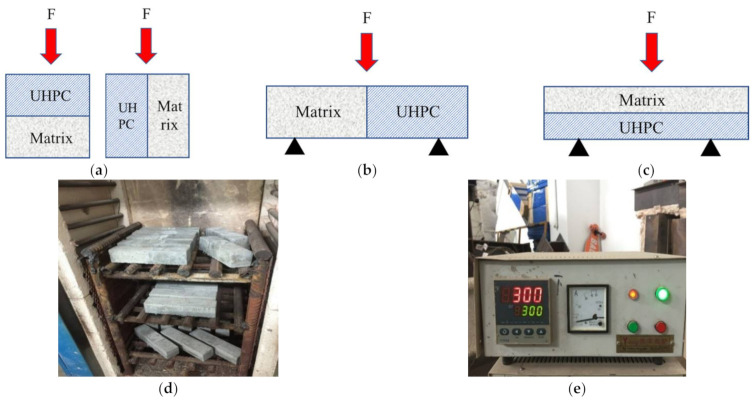
Sample preparation and high-temperature devices for the tests, where (**a**–**c**) represent the specimens utilized to assess the system’s compressive, bonding, and flexural performance, (**d**,**e**) represent the high temperature test furnace and its controller.

Figure 1a–c illustrates the different bonded samples for the mechanical tests. The ordinary cement-based matrix was cast and cured in advance according to the mix proportion in Table 3. Before casting the UHPC layer, the matrix was put into the corresponding mold. Following that, 100 mm × 100 mm × 100 mm cubic specimens were prepared to test the compressive strength. The prefabricated 50 mm thick matrix material was placed in the bottom of the cube mold, and the UHPC material was cast at the top without any surface preparation or vibration between two layers. Then, 40 mm × 40 mm × 160 mm prism specimens were prepared to test the bonding and flexural behaviors. For bonding strength tests, the left half of the prisms were precast matrices while they were at the bottom half for flexural behavior tests, and the other half of the specimens were UHPC material layers. No surface preparation or vibration was performed when casing the prism specimens as well.

## 3. Experimental Program and Methods

One day after casting, the specimens were demolded. Then, they were put into the standard curing room (temperature: 20 °C ± 2 °C, humidity ≥ 95%). One week before the high temperature test, the specimens were taken out and placed in the laboratory at room temperature (about 20 °C, T1), which was called the moisture removal treatment. When the repaired systems were subjected to the high-temperature environment, their age was 56 days.

The accessing methods for the effect of high temperature on the bonded cementitious composites system include:

1. High-temperature processing: It was carried out in a high-temperature furnace, as Figure 1d,e shows. The bonded systems were evenly placed in the high-temperature furnace, and the test temperatures were 100 °C (T2), 300 °C (T3), and 500 °C (T4). The heating rate was 5 °C/min. The target temperature was kept for half an hour. The specimens were taken out for subsequent testing when the temperature reduced to 20 °C. Based on the ISO 834 or ASTM E119 tests [24,25], the temperature experienced on the surface of cement-based composites may reach around 1200 °C. However, the temperature experienced within the bulk of the material—which is likely govern the performance of the structure—is typically notably smaller (below 1000 °C), based on its distance from the surface [26,27]. The thickness of the repair system is generally larger than 50 mm, at which point the temperature in the concrete specimen after 2 h in a fire was around 500 °C. Meanwhile, the thermal damage of concrete or fiber reinforced cementitious composites subjected to 500 °C was between 0.5 and 0.7 [28], which suggests the main degradation stage occurs at 500 °C. For these reasons, herein, the maximum temperature of 500 °C was employed.
2. Physical properties tests: The mass change was measured before and after the high-temperature test. Three specimens were tested as one group. Appearance change and explosive spalling property were recorded during the whole process.
3. Compressive strength tests: Bairoe electronic universal testing machine (Shanghai, China) was used to test the compressive strength, and the loading rate was 0.5 MPa/s. Two compression modes were adopted, i.e., the compression direction was parallel or perpendicular to the bonding surface (abbreviated as ∥ or ⊥).
4. Bonding/flexural strength tests: Three-point bending tests were carried out by an electronic universal testing machine controlled by a SANS microcomputer (Bairoe company, Shanghai, China), and its loading rate was 0.08 mm/min. In flexural tests, the mid-span deflection was read by an electronic extensometer (Central iron & steel research institute, Beijing, China) and the load-deflection curve was automatically recorded. The bonding strength was indirectly expressed by the bonding flexural strength, which was determined by the following Equation (1)
  *F* = 1.5*P* × *L/b^3^* = 2.34*P* (1)
  where *L* = 100 mm; *b* = 40 mm; *P* is the peak force; *F* is the bonding flexural strength of the system.
5. Microstructural analysis: The apparatus used for X-ray diffraction analysis (XRD) was a Rigaku Ultimate IV made in Tokyo, Japan with a scanning rate of 5°/min in the scanning range 5–75°. The 3H-2000PSI/2 specific area and pore size distribution analyzer produced by Beishide (Beijing, China) was used to analyze the pore structure of the specimens. The microstructure of the bonding area was analyzed using ZEISS195 ULTRA 55 type field emission scanning electron microscope (SEM) (Jena, Germany).

## 4. Experimental Results and Discussion

### 4.1. Physical Properties

#### 4.1.1. Appearance Changes and Explosive Spalling Property

**Figure 2:**
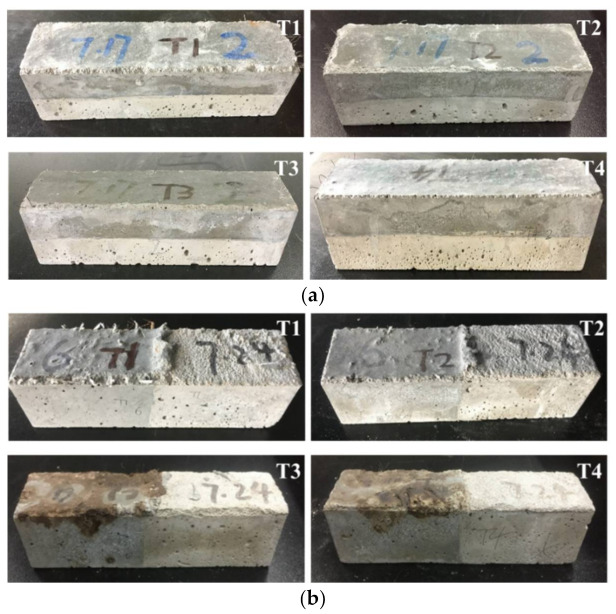
Appearance change of the repaired systems under different temperatures: (**a**) group 2 (Sf1), (**b**) group 4 (Sf1pp1).

The appearance change of the UHPC repaired systems at different temperatures, shown in Figure 2a, is the result for specimens of group 2 (Sf1) used for the flexural performance test; Figure 2b is the result for the specimens of group 4 (Sf1pp1) for the bonding performance test.

As Figure 2a shows, with the increase of temperature, both the UHPC and matrix layers changed to some extent. At room temperature, the UHPC layer was gray-black, and the matrix layer was gray. When the temperature rose to 100 °C, the appearance did not change much. When the temperature reached 300 °C, the color started to become lighter and a small number of micro cracks could be observed on the surface of the system. For the temperature of 500 °C, the bonded system began to turn yellow. A few micro cracks appeared on the surface of the system. As for group 4 with PP fibers, it can be observed from Figure 2b that with the increase of temperature, its appearance changed greatly, especially at 300 °C and 500 °C. This is mainly because the melting point of PP fiber is about 160–170 °C. When the heating temperature is higher than its melting point, the PP fiber inside the UHPC layer melts and forms pores. The one close to the surface will become yellow or even black after directly subjected to high temperature, which is similar to the phenomenon of organic burning.

For the repaired systems prepared in this paper, both the plain UHPC group (group1) and the single-doped steel fiber group (group 2, 3) had explosive spalling phenomenon during the high temperature test. For the Sf0 group, the first explosion occurred when the heating temperature was close to 300 °C, accompanied by a “bang” sound. With the continuous rise of temperature, several more bursts occurred. As for Sf1 and Sf2 groups with steel fibers, even though there was a slight “bang” sound when the temperature exceeded 400 °C, the overall integrity of the system was much better than the plain UHPC repaired group. Only a small number of edges or corners fell off at the end of the high-temperature test. This may be because with the increase of the steel fiber content, the cohesive force inside the UHPC increases, thus reducing the explosive spalling degree of the system [29].

For system with PP fibers, their high-temperature bursting resistance was better than that of groups 1–3. Most of the specimens with PP fibers withstood the high temperature of 500 °C, and a very small number of them burst slightly near 500 °C or at the constant temperature stage. It is generally believed the PP fiber can be uniformly and randomly distributed in the cementitious composites, the pores left by the melting and volatilization of PP fiber can also be considered to be evenly distributed, which is beneficial for the discharge of heat and water vapor generated in UHPC under high temperatures, thus, reducing the internal pore pressure and improving the explosive spalling property of UHPC [30]. Similar conclusions have also been drawn by Sun et al. [31] and Dong et al. [32].

#### 4.1.2. Mass Change

**Figure 3:**
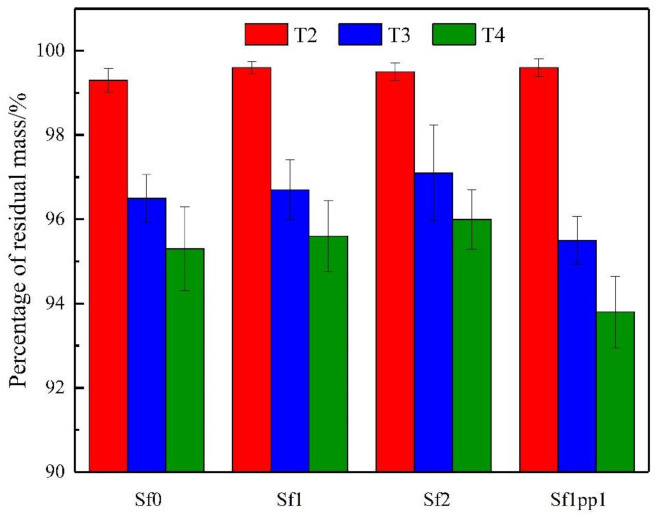
Percentage of residual mass of the repaired systems under high temperatures.

The mass change of each bonded group under high temperature is shown in Figure 3. When the heating temperature was 100 °C, the percentage of residual mass of all repaired groups was about 99.5%, which was almost negligible. At this point, it could be considered that the decrease in mass was caused by the evaporation of free water in both matrix and UHPC layers after high temperatures. When the heating temperature reached 300 °C, the mass of all repaired systems showed a relatively obvious decline. The average percentage of residual mass was about 96%. In particular, group 4 with 1% PP fiber had the largest mass loss due to the melting of PP fibers at this temperature, and its mass residual percentage was only 95.5%. After exposure to the temperature of 500 °C, the percentage of residual mass showed a further decrease, with an average of 94–95%. At this stage, the mass loss of the repaired systems included not only the dehydration and decomposition of the C-S-H gel but also the thermal decomposition of Ca(OH)_2_, CaCO_3_, etc. [33], which led to a significant decrease in mass again. By comparing the bonded systems of groups 1–4, it can be noted that the system with PP fibers still had a greater mass loss due to the melting of fibers, while groups 1–3 without PP fibers had higher percentages of residual mass at the temperature of 500 °C.

### 4.2. Compressive Strength

**Figure 4:**
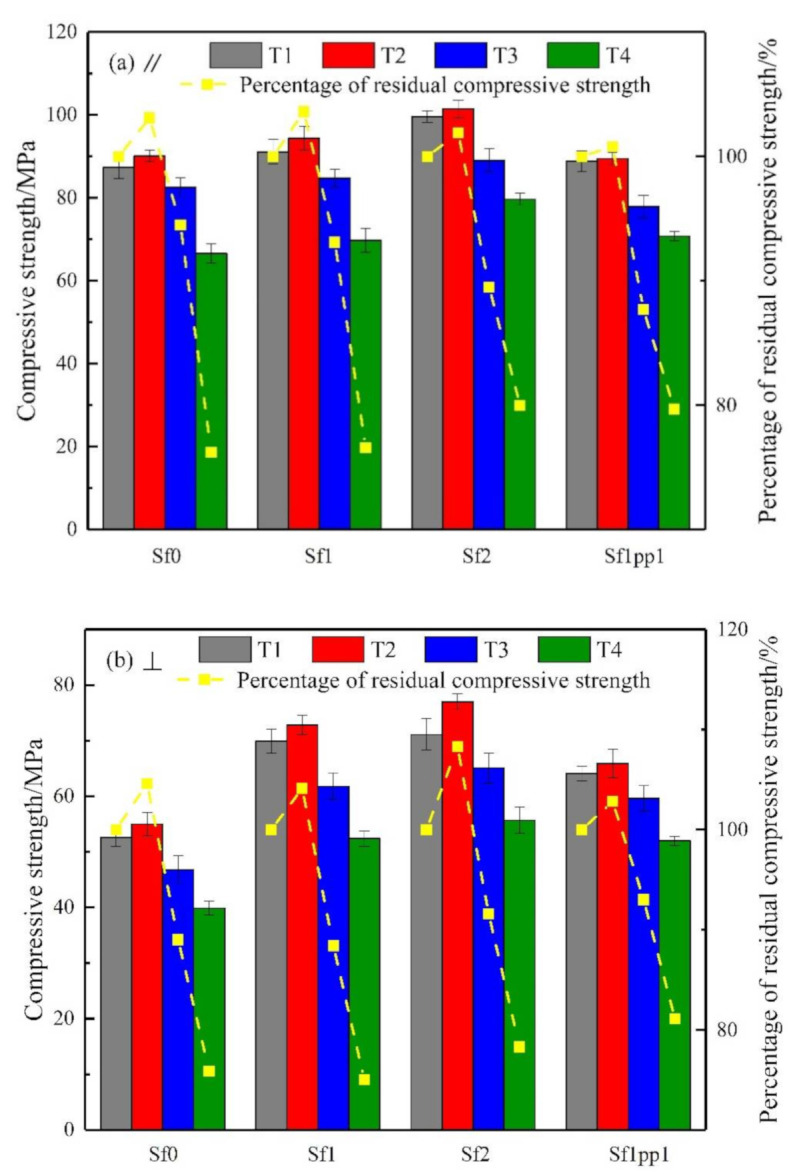
Evolution and residual percentage of compressive strength of the repaired systems under high temperatures: stress direction (**a**) ∥, (**b**) ⊥.

Figure 4 shows the changes in compressive strength and its residual percentage at room temperature, 100 °C, 300 °C, and 500 °C of UHPC repaired systems. For Sf0, Sf1, and Sf2 systems, in the range of room temperature −100 °C, the compressive strength showed a small increase, with an average of 5%, which was consistent with the conclusions of Zheng et al. [34] and Deshpande et al. [35] on the improvement of compressive strength of RPC at the initial stage of high-temperature. This is mainly because the UHPC material is mixed with mineral admixtures such as silica fume, which can cause the pozzolanic reaction and cement hydration reaction more fully at a temperature of about 100 °C. Therefore, it could be regarded as a “high-temperature curing” process at this time, and the compressive strength improved slightly. When the temperature rose to 300 °C, the compressive strength under both stress modes decreased to a certain extent. For example, the decrements were 5.5%, 6.9%, 10.5% (∥) and 11.0%, 11.5%, 8.4% (⊥) for Sf0, Sf1, and Sf2 groups, respectively, compared to the room temperature conditions. The slight decrease in strength during this period is caused by the evaporation of the original free water inside the system and the generation of microcracks [34]. Under the action of load, stress concentration occurs at the tip of the microcrack, which causes the further development of the microcrack [35]. When they were exposed to the temperature of 500 °C, the compressive strength was significantly degraded, which could be reduced by about 20%. In this case, it was also the result of the dehydration of C-S-H gel and the thermal decomposition of Ca(OH)_2_ and CaCO_3_, as mentioned in Section 4.1.2 [33]. Meanwhile, it can also be seen that with the addition of steel fiber, the compressive strength of the bonded system at all temperatures increased to a certain extent, which is not only related to the strengthening and toughening effect of steel fiber but also related to its good heat transfer property, which was also observed by Zhang et al. [36]. Therefore, the difference between internal and external temperature of the system was not too large when exposed to high temperature, thus, reducing the temperature stress and improving the compressive strength of the system. For the hybrid fiber system Sf1pp1, the evolution of high temperature performance was similar to that of groups 1–3, and the compressive strength also showed a slight increase first, followed by a decrease.

### 4.3. Bonding Strength

**Figure 5:**
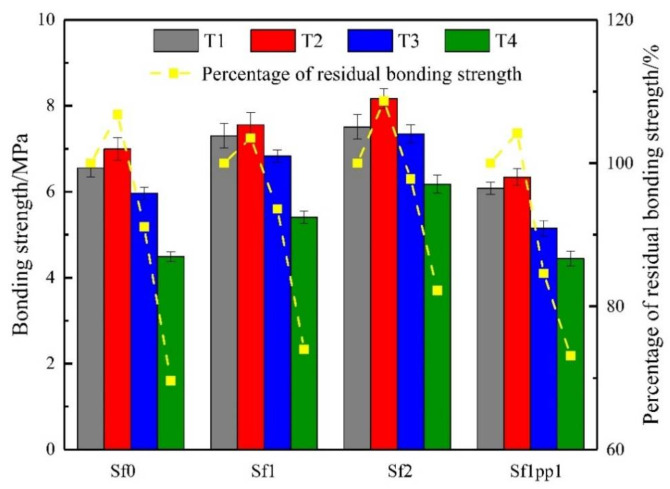
Evolution and residual percentage of bonding strength of the repaired systems under high temperatures.

The test results of bonding strength and its residual percentage are exhibited in Figure 5 for a UHPC repaired system under high temperature. For the Sf0, Sf1, and Sf2 repaired systems, it can be seen from Figure 5 that whether it was at room temperature or high temperature, the addition of steel fibers could effectively improve the bonding strength. The system with 2% steel fiber content had the best bonding performance. This improvement mainly comes from the enhancement of the mechanical occlusal effect generated from the steel fibers at the interface [37] and the restriction effect on the dry shrinkage deformation and shrinkage stress of the fibers [38]. When the temperature was 100 °C, the bonding strength of the system was slightly improved. The residual percentages of the bonding strength of the Sf0, Sf1, and Sf2 groups were 106.8%, 103.5%, and 108.7%, respectively, with an average improvement of about 6%. This statement agreed with the conclusions of Fernandes et al. [39]. When the temperature rose to 300 °C, the bonding strength decreased slightly compared to the room temperature group. The percentage of residual bonding strength for Sf0, Sf1, and Sf2 groups were 91.1%, 93.6%, and 97.8%, respectively. It seems that the addition of steel fibers can resist the degradation of bonding performance of the systems at high temperature to some extent. When the heating temperature reached 500 °C, the bonding strength of Sf0 group dropped sharply, with only 68.6% residual percentage, while the Sf1 and Sf2 groups were 74.0% and 82.2%. At this time, the degradation of bonding performance was mainly caused by the failure of hydration products with cementing effects after being heated, and the results also proved once again that the addition of steel fiber could effectively improve the bonding performance of the repaired systems at high temperatures. For the hybrid fiber system Sf1pp1, the evolution trend of the bonding strength of the hybrid fiber system was similar to that of single-mixed fiber groups.

### 4.4. Flexural Behavior

#### 4.4.1. Failure Mode

**Figure 6:**
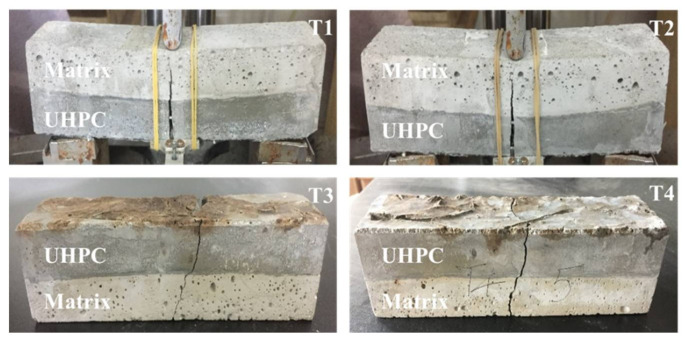
Failure modes of the UHPC repaired systems under different temperatures (Sf1pp1 group).

When exposed to high temperature, different groups of UHPC repaired systems showed different failure modes. For Sf1, Sf2 and Sf1pp1 groups, the failure mode was that micro cracks first appeared in the UHPC layer in the tensile zone. With the increase of load, the cracks expanded upward and gradually reached the bonding surface. Eventually the cracks ran through the whole bonded system. The specimens could still bear the load after cracking, showing obvious characteristics of ductile failure. Whereas for the Sf0 group without steel fiber, brittle failure occurred at all temperatures. The system exhibited a sudden brittle failure, accompanied by a loud noise. The bonded system was directly destroyed into two halves. For all bonded systems, the steel fibers and PP fibers with bridging effect could be observed at the crack, as presented in Figure 6.

#### 4.4.2. Load-Deflection Response

**Figure 7:**
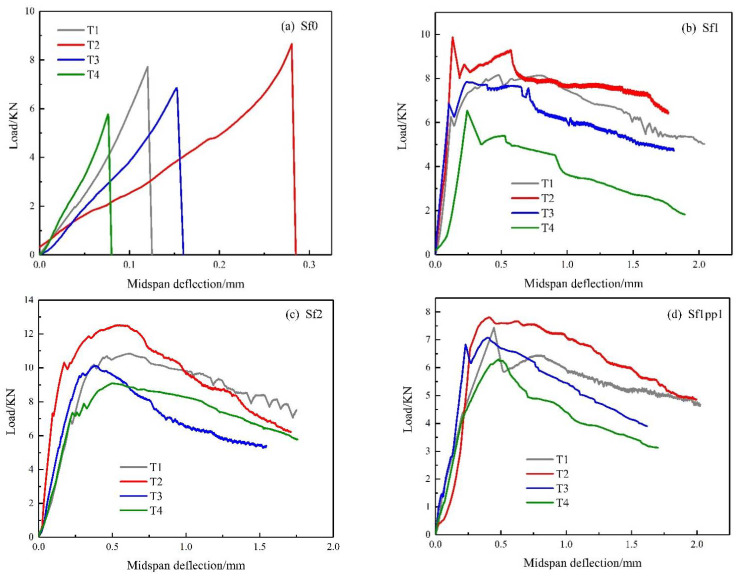
Load-deflection curves of each repaired system at different temperatures: (**a**) Sf0; (**b**) Sf1; (**c**) Sf2; (**d**) Sf1pp1.

Figure 7 presents the load-deflection curves of each repaired system at different temperatures. Figure 7a–c displays the load-deflection response of the Sf0, Sf1, and Sf2 systems. It is obvious that when steel fibers were added, the flexural capacity of the system was significantly improved, and the failure mode also showed a great change. For the Sf0 group, the shape of the load-deflection response was basically the same from room temperature to 500 °C. Both the maximum load and the midspan deflection increased to some extent from room temperature to 100 °C. The maximum load at 100 °C was about 1.1 times that at room temperature, and the corresponding deflection was about two times the initial value. It can be seen that an appropriate high temperature was beneficial to the toughness of the systems. However, with the continuous increase of temperature, the flexural capacity of the systems decreased, and the toughness became worse. Sf1 and Sf2 systems also showed the maximum flexural capacity at 100 °C and then deteriorated with the increase of temperature. In addition, when exposed to the temperature of 500 °C, the load-deflection response of Sf1 group decreased to a certain extent after the initial crack. Although it was still able to bear the load, the flexural performance was always less than the initial crack load, whereas the Sf2 group could still maintain a high load bearing capacity, which reflected that the Sf2 group had better resistance to high temperature than the Sf1 one. For the hybrid fiber system, as Figure 7d shows, when exposed to the temperature of 100 °C, the maximum load of the system increased by about 5% compared to the room temperature groups. The degradation degree of flexural capacity was very low under the action of high temperature, and the decrements were 5.1% and 15.6% for the temperature of 300 °C and 500 °C, respectively.

### 4.5. Microstructural Analysis

#### 4.5.1. XRD Analysis

**Figure 8:**
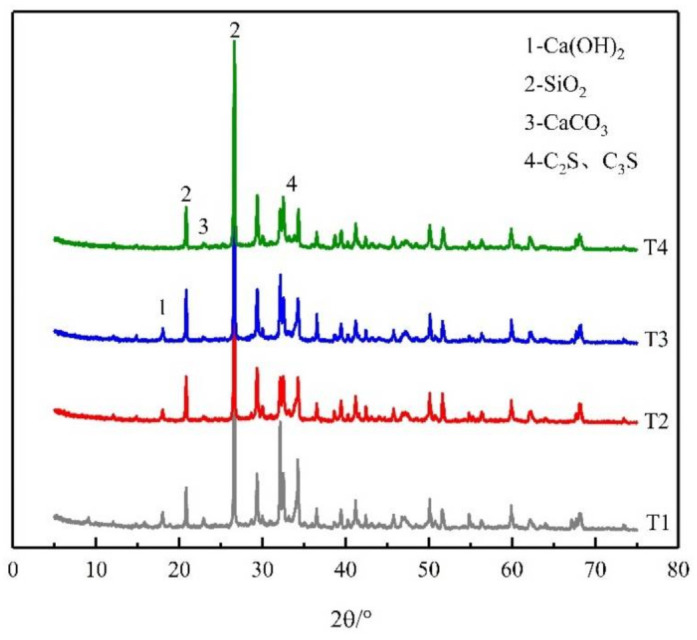
XRD characteristic peaks of UHPC repair materials at different temperatures.

Figure 8 shows the XRD characteristic peaks of UHPC repair materials at different temperatures. It was found that with the increase of fire temperature, the peak values of the original components in UHPC were mostly reduced with varying degrees. For the component of Ca(OH)_2_, the characteristic peak did not change much when the temperature was lower than 300 °C. However, when it reached 500 °C, the diffraction peak of Ca(OH)_2_ could not be detected any more, which indicates that the Ca(OH)_2_ component had been completely decomposed at this temperature [33]. As for C_2_S and C_3_S, when the heating temperature rose from room temperature to 100 °C, the diffraction peak intensity decreased significantly. This is the result of the promotion effect on hydration process of the unhydrated cement particles at a suitable high temperature, which confirms the “high-temperature curing” process mentioned above [34,35].

#### 4.5.2. BET Analysis

**Figure 9:**
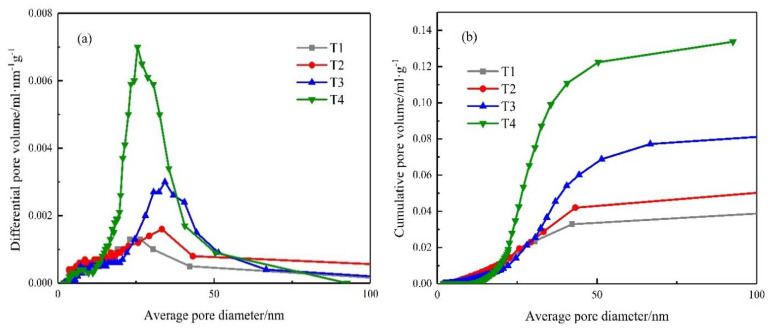
BET analysis of UHPC repair materials: (**a**) differential pore volume, (**b**) cumulative pore volume.

Figure 9 shows the differential pore volume and cumulative pore volume distribution of the UHPC repair material obtained by the BET test. In general, with the increase of heating temperature, the pore volume of UHPC repair materials increased significantly. This statement is consistent with the findings made by Wu [40], who studied the effect of high temperature on microstructure and vapor pressure of high performance concrete. This may be because when the temperature reached 100 °C, the free water in UHPC evaporated. As a result, the pore volume increased slightly at this time. With the continuous increase of heating temperature, the bound water evaporated, and micro cracks began to appear in the concrete [17]. The loss of bound water of the hydrated compounds of the cement caused a strong weakening of the material that involved the appearance of fissures and a considerable increase in porosity [41].

When the temperature reached 500 °C, the Ca(OH)_2_ in UHPC decomposed, and the cracks grew rapidly, leading to the deterioration of UHPC pore structure [40]. When the average pore diameter was in the range of 0–100 nm, the cumulative pore volume of UHPC at 500 °C could reach more than three times that of room temperature.

#### 4.5.3. Scanning Electron Microscope Observations

**Figure 10:**
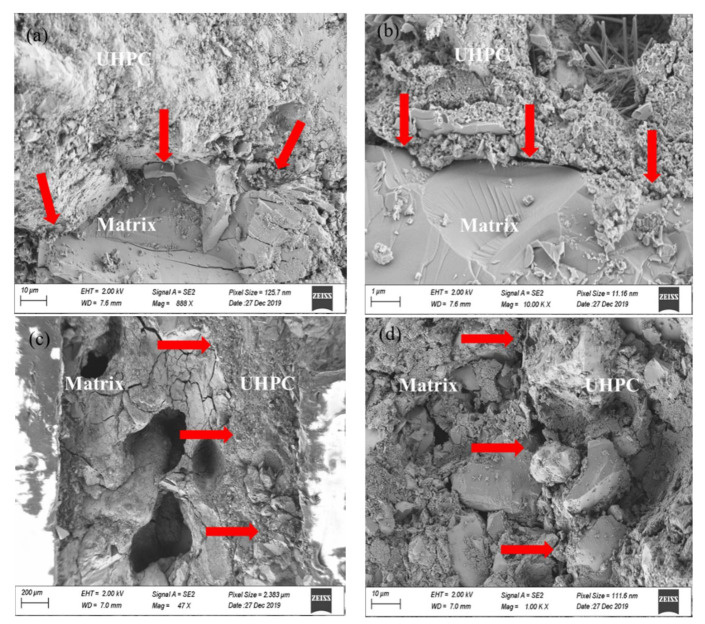
SEM micrographs of the UHPC repaired system: (**a**,**b**) T1, (**c**,**d**) T4 (arrows: bond area).

Figure 10 illustrates the SEM micrographs of the bonding interface at room temperature and 500 °C. It was found that there were obvious bonding joints between the matrix and UHPC layer at different temperatures. For the repaired systems at room temperature, the upper part in Figure 10a,b was the UHPC phase, and the other part was the matrix. Gels were abundant in the UHPC phase and were the main source of strength, while a large number of Ca(OH)_2_ crystals and other substances could be observed in the matrix. After experiencing a high temperature of 500 °C, the morphology of the bonding area was obviously deteriorated, and the hydration products were greatly reduced. Substances such as Ca(OH)_2_ and AFt could hardly be found. This is mainly because AFt decomposed at about 200 °C and Ca(OH)_2_ also decomposed into CaO and water at around 500 °C [17]. As shown in Figure 10c, at 500 °C, a large number of holes appeared in the matrix part on the left. Although the UHPC layer was also deteriorated to some extent, the integrity of the overall morphology was obviously better than that of the matrix part, which indicates that the UHPC repair material layer can withstand the high temperature of 500 °C well. According to Figure 10d, after exposure to the temperature of 500 °C, the bonding area of the repaired system also showed obvious deterioration, and the interface structure became very loose. This is exactly the main reason why the bonding strength of the system decreased significantly after the high temperature of 500 °C.

From the above, the degradation of the mechanical performance of the UHPC bonded system can be explained by the decomposition of the components, the deterioration of the UHPC’s pore structures, and the microstructures of the repairing interface. In addition, the deterioration of the interfacial bonding between the steel fibers and the UHPC matrix is also an important reason for the degradation of the mechanical performances at high temperatures [36]. According to Zhang et al. [36], the peak pull-out force and average bonding strength decrease significantly when temperature is above 300 °C. When the temperature reaches 600 °C, the average bonding strength losses are more than 60%.

## 5. Conclusions

The effect of various high temperature levels on the mechanical behaviors of the UHPC repaired cementitious composites are investigated in this paper. The following conclusions can be drawn from the experimental results:

(1) After exposure to 500 °C, UHPC repaired composites change to light yellow from gray at ambient temperature and visible cracks can be observed on the surface.
(2) After heating to 500 °C, the average percentage of residual mass of the system is around 94–95%, and UHPC with hybrid fibers has the best high temperature explosive spalling resistance.
(3) The residual compressive strength, bonding strength, and flexural capacity exhibit a slight increase at 100 °C but then decrease with the heating temperature. For example, after heating to 500 °C, the residual compressive strength, bonding strength, and flexural capacity decrease by about 20%, 30%, and 15%, respectively. Moreover, the addition of fibers can help improve the mechanical performance of UHPC repaired composites.
(4) Based on the microstructural observation, it is found that dehydration of the hydrate products, cracking, and coarsening of the pores result in the loose microstructure of the interfacial transition zone. Ultimately, the bonding performance deteriorates.

## Figures and Tables

**Table 1:** Chemical components of cement (wt, %).

Oxides	CaO	MgO	Al_2_O_3_	SiO_2_	P_2_O_5_	SO_3_	K_2_O	Na_2_O	TiO_2_	Cr_2_O_3_	MnO	Fe_2_O_3_	CuO	ZnO	SrO
Content	65	0.65	4.56	20.90	0.12	2.65	0.87	0.08	0.22	0.01	0.09	3.23	0.01	0.05	0.03

**Table 2:** Properties of fibers.

Type of Fibers	Diameter (mm)	Length (mm)	Density (g/cm^3^)	Tensile Strength (MPa)	Elastic Modulus (GPa)
Steel fibers	0.2	13	7.85	2850	200
PP fibers	0.1	40	0.91	600	10

**Table 3:** Basic mix proportion of materials (kg/m^3^).

Material	Cement	Silica Fume	Quartz Powder	Quartz Sand	Sand	Superplasticizer	Water
UHPC	743	90	250	1070		7.15	193
Matrix	500				1500	0.6	220

**Table 4:** Key components of UHPC layer for each repaired system group.

Group	Category	Steel Fiber (%)	PP Fiber (%)	28d Compressive Strength (MPa)
1	Sf0			131.2
2	Sf1	1		155.9
3	Sf2	2		164.4
4	Sf1pp1	1	1	150.6

## Data Availability

The data presented in this study are available on request from the corresponding author. The data are not publicly available science they would be one part of our ongoing study.
